# Actions in Support of Newborn Screening for Critical Congenital Heart Disease — United States, 2011–2018

**DOI:** 10.15585/mmwr.mm6805a3

**Published:** 2019-02-08

**Authors:** Jill Glidewell, Scott D. Grosse, Tiffany Riehle-Colarusso, Nelangi Pinto, Jeff Hudson, Rachel Daskalov, Amy Gaviglio, Erin Darby, Sikha Singh, Marci Sontag

**Affiliations:** ^1^National Center on Birth Defects and Developmental Disabilities, CDC; ^2^American Academy of Pediatrics, Itasca, Illinois; ^3^Minnesota Department of Health; ^4^Association of Public Health Laboratories, Silver Spring, Maryland; ^5^Colorado School of Public Health, University of Colorado, Aurora, Colorado.

In 2011, the U.S. Department of Health and Human Services added critical congenital heart disease (CCHD), which occurs in two of every 1,000 births, to the list of conditions recommended to states for universal newborn screening (*1*). Without early detection, infants with CCHD are at risk for substantial morbidity and death in the first weeks and months of life (*2*). Based on 2007–2013 data, deaths from CCHD and other cardiac causes in infants aged <6 months significantly declined in infants born in eight states after they had fully implemented mandated newborn CCHD screening policies by June 2013 (*3*). CDC collaborated with the American Academy of Pediatrics (AAP) and the Association of Public Health Laboratories’ Newborn Screening Technical Assistance and Evaluation Program (NewSTEPs) to update a 2015 report (*4*) on states’ actions toward adopting and implementing policies supporting CCHD newborn screening. In 2018, all 50 states and the District of Columbia (DC) had implemented CCHD screening policies, and, with one exception, all states mandated that screening be done (California mandates that screening be offered). However, not all states had data systems in place for tracking all screening results and outcomes. Ongoing evaluation activities, which rely on screening data, could help identify program improvement opportunities and monitor the impact of early identification of CCHD.

Congenital heart defects occur in approximately eight of every 1,000 live births; one fourth of infants born with congenital heart defects have CCHD (*1*,*2*). CCHD typically requires surgical or catheter intervention before age 1 year (*2*). Newborn screening can identify newborns with CCHD before signs or symptoms are evident and before hospital discharge after birth. CCHD screening supplements clinical detection of CCHD to facilitate timely identification, treatment, and management of affected infants. Infants are screened for CCHD using pulse oximetry, a noninvasive method to estimate the oxygen saturation in an infant’s arterial blood. Hypoxemia (abnormally low oxygen saturation) detected by pulse oximetry screening can result from CCHD or other causes. Additional testing (e.g., chest radiograph or echocardiography) is needed after an abnormal screen to determine the cause of the hypoxemia (*2*,*5*,*6*).

CDC, AAP, and NewSTEPs assessed actions by states (i.e., legislation, regulations, or both) toward adoption and implementation of policies supporting CCHD newborn screening. In the context of this report, a statute is a law enacted by a state legislature and signed into law, a regulation is considered to be a rule promulgated by a state agency with the force of law, and legislation is a bill reviewed and acted upon by a state legislature. Policies include statutes, regulations, and other measures, such as appropriations. The effective date of a statute can differ from the date it is implemented by health care providers. For example, Maryland enacted a screening mandate in May 2011 that legally took effect in July 2011 (*4*). However, the effect of the statute was to direct the state health department to begin the process of preparing regulations that, once issued, would require hospitals and other delivery care providers to screen for CCHD. The date on which the Maryland screening mandate was actually implemented at the provider level was September 1, 2012 (*3*). In this report, the implementation date is the date when providers were expected or required to begin universal screening of newborns for CCHD.

AAP and NewSTEPs used several methods to gather and compile enactment, effective, and implementation dates of screening policies, as well as information on screening data collection and data sharing. AAP monitored state legislation using legal and regulatory tracking software and researched regulatory and hospital guidelines on state websites. AAP obtained primary information through direct contact and partnership with AAP state chapters. State-specific information on collection of screening data elements was provided by state CCHD screening programs directly to the NewSTEPs Data Repository (*7*). NewSTEPs surveyed state CCHD newborn screening coordinators to assess data sharing and collaboration between birth defects surveillance programs, which track cases of CCHD, and newborn screening programs. Newborn screening programs in all 51 jurisdictions (50 states and DC) participated in the survey.

From 2013 to 2018, the number of jurisdictions that had implemented CCHD screening policies increased from 22 to 51 (Table 1). States used various approaches to adopt newborn screening for CCHD. Thirty-nine (76%) jurisdictions adopted statutes that either mandated screening or the offer of screening or called for the issuance of regulations to mandate that screening be offered; the other 12 jurisdictions implemented mandates exclusively through regulations. The content of policies varies among states. For example, in 2015, Colorado mandated that infants born in a birthing center located below 7,000 feet elevation be screened for CCHD (infants born at higher-elevation locations typically have lower normal oxygen saturation levels, which have not yet been incorporated in screening guidelines). One year later, the state required midwives attending home births to either screen newborns or refer the parents to a physician or health facility. Kansas, which previously had a successful voluntary CCHD screening project in place since 2013, added CCHD to its required newborn screening panel by regulation in early 2018. In Idaho, regulations went into effect in July 2018 that require all newborns to be screened for CCHD, including those born outside of a birthing center or hospital.

**TABLE 1:** State legislation and regulations for newborn screening for critical congenital heart disease (CCHD) — United States, 2011–2018

State	Citation	Statute*	Regulation/Guidance^†^	Actions	Date enacted	Date effective	Date universal screening policy implemented^§^
Alabama	Ala. Admin. Code 420–10–1	—	X^¶^	Mandates screening	May 2013	Jun 2013	Jun 21, 2013
Alaska	Alaska Stat § 18.15.205	X^¶^	—	Mandates screening	Sep 2013	Jan 2014 (Jan 2016 for providers who attend <20 births/yr)	Mar 19, 2014
	Alaska Admin. Code tit. 7, § 27.630 Alaska Admin. Code tit. 7, § 27.635	—	X	Specifies type of provider who is required to perform screen; reporting requirements	Feb 2014	Mar 2014	
Arizona	Ariz. Rev. Stat. § 36–694	X^¶^	—	Mandates screening	Apr 2014	Jul 2014	Jul 1, 2015
	Ariz. Admin. Code § R9–13–202	—	X	Screening and reporting requirement	May 2015	Jul 2015	
Arkansas	Ark. Code Ann. § 20–9-13	X^¶^	—	Mandates screening	Apr 2013	Aug 2013	Jul 1, 2015
California	Cal. Hsc. Code § 124121	X	—	Mandates screening be offered	Sep 2012	Jan 2013	Jul 1, 2013
Colorado	Colo. Rev. Stat. § 25–4-1004.3	X^¶^	—	Mandates *s*creening in birthing facilities below 7,000 ft. altitude	May 2015	Aug 2015	Jan 1, 2016
	Colo. Rev. Stat. § 12–37–105	X	—	Mandates direct entry midwives perform screen**	Jun 2016	Aug 2016	
Connecticut	Conn. Gen. Stat. § 19a-55	X^¶^	—	Mandates screening	May 2012	Jan 2013	Jan 1, 2013
Delaware	16 Del. Admin. Code § 4107.4	—	X^¶^	Mandates screening	May 2013	May 2013	May 1, 2013
District of Columbia	D.C. Code § 7–857.02	X^¶^	—	Mandates screening	Jun 2015	Sep 2015	Sep 7, 2015
Florida	Fla. Admin. Code r. 64C-7.002	—	X^¶^	Mandates screening	Oct 2014	Oct 2014	Mar 26, 2015
Georgia	Ga. Comp. R. and Regs. 511–5-5-.03	—	X^¶^	Mandates screening	May 2014	Jun 2014	Jul 1, 2015
Hawaii	Haw. Rev. Stat. § 321–296	X^¶^	—	Mandates screening	Jul 2015	Jul 2015	Jan 2014
Idaho	Idaho. Admin. Code. r. 16.02.12.301	—	X^¶^	Mandates screening	Jul 2018	Jul 2018	Jul 1, 2018
Illinois	410 Ill. Comp. Stat. § 240/1.10	X^¶^	—	Mandates screening	Aug 2013	Aug 2013	Aug 20, 2013
Indiana	Ind. Code § 16–41–17–2	X^¶^	—	Mandates screening	May 2011	Jan 2012	Jan 1, 2012
Iowa	Iowa Code § 136A.5A	X^¶^	—	Mandates screening	Jun 2013	Jul 2013	Jan 8, 2015
	Iowa Admin. Code r. 641.4.3	—	X	Screening and reporting requirements	Dec 2014	Jan 2015	
Kansas	Kan. Admin. Regs. § 28–4-502	—	X^¶^	Mandates screening	Feb 2018	Feb 2018	Feb 2018
Kentucky	Ky. Rev. Stat. Ann. § 214.155	X^¶^	—	Mandates screening	Mar 2013	Jan 2014	Jan 1, 2014
	902 Ky. Admin. Regs. 4:030	—	X	Screening and reporting requirements	Dec 2013	Dec 2013	
Louisiana	La. Stat. Ann. § 40:1083.3	X^¶^	—	Mandates screening	Jun 2013	Aug 2013	Aug 1, 2013
Maine	Me. Stat. tit. 22, § 1532	X^¶^	—	Mandates screening	Jul 2013	Jul 2013	Oct 9, 2013
	10–144 Me. Code. R. 709	—	X	Screening and reporting requirements	Sep 2015	Sep 2015	
Maryland	Md. Code, Health. Law § 13–111	X^¶^	X	Mandates screening and creates advisory committee to develop implementation recommendations	May 2011	Jul 2011	Sep 1, 2012
	Md. Code Regs. 10. 52.15.01-.08	—	X	Screening and reporting requirements	Oct 2012	Oct 2012 (emergency adoption) Apr 2013 (permanent adoption)	
	Md. Code, Bus. and Occ. Law § 8–6C-2	X^¶^	—	Mandates direct entry midwives** perform screen	May 2015	Jun 2015	
Massachusetts	Mass. Gen. Laws ch. 111, § 110C	X^¶^	—	Mandates screening	Mar 2014	Jun 2014	Jun 2014
	105 Code Mass. Regs. 142.303	—	X	Requires freestanding birth centers to develop screening protocols	Oct 2014	Jan 2015	
	105 Code Mass. Regs. 130.616	—	X	Requires hospitals to develop screening protocols	Oct 2014	Jan 2015	
Michigan	CCHD mandate letter to hospital administrators (authority under Mich. Comp. Laws § 333.5431)	—	X^¶^	Mandates screening	Oct 2013	Apr 2014	Apr 1, 2014
Minnesota	Minn. Stat. § 144.1251	X^¶^	—	Mandates screening	May 2013	Aug 2013	Aug 1, 2013
Mississippi	Miss. Code R. § 15.4.1.1	—	X^¶^	Mandates screening	Oct 2014	Nov 2014	Jul 1, 2015
Missouri	Mo. Rev. Stat. § 191.334	X^¶^	—	Mandates screening	Jul 2013	Aug 2013	Jan 1, 2014
Montana	Mont. Admin. R. 37.57.305	—	X^¶^	Mandates screening	Jun 2014	Jul 2014	Jul 1, 2014
Nebraska	Neb. Rev. Stat. § 71–556	X^¶^	—	Mandates screening	Jun 2013	Sep 2013	Sep 6, 2013
	181 Neb. Admin. Code 10	—	X	Screening requirements	Aug 2014	Aug 2014	
Nevada	Nev. Rev. Stat. § 442.680	X^¶^	—	Mandates screening	Jun 2013	Jul 2015	Jul 2015
New Hampshire	N.H. Rev. Stat. Ann. § 132:10-aa	X^¶^	—	Mandates screening	Jun 2012	Aug 2012	Aug 11, 2012
New Jersey	N.J. Rev. Stat. § 26:2–111.4	X^¶^	—	Mandates screening	Jun 2011	Aug 2011	Aug 31, 2011
	N.J. Code Admin. § 8:43G-19.15	—	X	Reporting requirements	Dec 2013	Jan 2014	
New Mexico	N.M. Stat. § 24–1-6	X^¶^	—	Mandates screening	Mar 2014	May 2014	Jul 1, 2014
New York	N.Y. P.B.H. Law § 2500-A	X^¶^	—	Mandates screening	Jul 2013	Jan 2014	Jan 27, 2014
North Carolina	N.C. Gen. Stat. § 130A-125	X^¶^	—	Mandates screening	May 2013	May 2013	Jul 25, 2014
	10 N.C. Admin. Code 43K.0102–0103	—	X	Screening and reporting requirements	Jul 2014	Jul 2014 (temporary effective date) Apr 2015 (permanent effective date)	
North Dakota	N.D. Cent. Code § 25–17–06	X^¶^	—	Mandates screening	Apr 2013	Aug 2013	Aug 2013
Ohio	Ohio Rev. Code § 3701.5010	X^¶^	*—*	Mandates screening	Jun 2013	Sep 2013	Oct 1, 2014
	Ohio Admin. Code 3701:54	—	X	Reporting requirements	Jun 2014	Oct 2014	
Oklahoma	Okla. Stat. tit. 63, § 1–550.5 Okla. Admin. Code § 310:550	X^¶^	—	Mandates screenin	Apr 2013	Jul 2013	Jul 1, 2013
	Okla. Admin. Code § 310:550	—	X	Screening and reporting requirements	Jun 2014	Sep 2014	
Oregon	Or. Rev. Stat. § 433.318	X^¶^	—	Mandates screening	Jun 2013	Jun 2013	Mar 1, 2014
	Or. Admin. R. 333–520–0060	—	X	Screening requirements	Dec 2013Jun 2014	Jan 2014 (temporary effective date) Jun 2014 (permanent effective date)	
Pennsylvania	42 Pa. B. 7348	—	X	Mandates reporting if screening is performed	Dec 2012	Mar 2013	Sep 2014
	Act of Jul. 2, 2014, P.L. 853, No. 94	X^¶^	—	Mandates screening	Jul 2014	Sep 2014	
Rhode Island	216 R.I. Code R. § 20–05–01	—	X^¶^	Mandates screening	Aug 2014	Jul 2015	Jul 1, 2015
South Carolina	S.C. Code Ann. § 44–37–70	X^¶^	—	Mandates screening	Jun 2013	Sep 2013	Sep 11, 2013
	S.C. Code Regs. 61–123	—	X	Screening requirements	Jun 2014	Jun 2014	
South Dakota	S.D. Codified Laws §34–24–32	X^¶^	—	Mandates screening	Mar 2013	Jul 2013	Jul 2013
Tennessee	Tenn. Code Ann. § 68–5-507	X	—	Creates advisory committee to develop screening program	Mar 2012	Jan 2013	May 31, 2013
	Tenn. Comp. R. and Regs. 1200–15–01	—	X^¶^	Mandates screening	May 2013	May 2013	
Texas	Tex. HSC. Code § 33.011	X^¶^	—	Mandates screening	Jun 2013	Sep 2013	Aug 7, 2014
	Tex. Admin. Code § 37.78-.79	—	X	Screening and reporting requirements	Jul 2014	Aug 2014	
Utah	Utah Code § 26–10–6	X^¶^	—	Mandates screening	Mar 2013	Oct 2014	Oct 1, 2014
Vermont	18 Vt. State. Ann. § 5087	X	—	Requires screening rules be issued	May 2016	Jul 2016	Dec 2016
	13 Vt. Code R. 140 057	—	X^¶^	Mandates screening	Dec 2016	Dec 2016	
Virginia	Va. Code Ann. § 32.1–65.1	X^¶^	—	Mandates screening	Feb 2014/Mar 2014	Jul 2014	Jan 1, 2015
	12 Va. Admin. Code § 5–71–30/12 Va. Admin. Code § 5–71–210	—	X^¶^	Screening and reporting requirements	Aug 2016	Oct 2016	
Washington	Wash. Rev. Code § 70.83.090	X^¶^	—	Mandates screening	Apr 2015	Jul 2015	Jul 24, 2015
West Virginia	W. Va. Code § 16–44–2	X^¶^	—	Mandates screening	Apr 2012	Jun 2012	Sep 1, 2012
Wisconsin	Wis. Stat. § 253.13	X	—	Allows the state’s department of health to add conditions to the state’s screening panel of disorders	Mar 2014	Mar 2014	Jul 3, 2014
	Wis. Admin. Reg. Em. Rule 1410	—	X^¶^	Mandates screening	Jun 2014	Jul 2014 (emergency effective date)	
	Wis. Admin. Code DHS § 115	—	X^¶^	Mandates screening	Jul 2015	Aug 2015 (permanent effective date)	
Wyoming	Wyo. Code R. § 048.0035.1.09072017	—	X^¶^	Mandates screening	Sep 2017	Sep 2017	Sep 7, 2017

**Abbreviation:** X = presence of state action.

* Thirty-nine states and the District of Columbia (DC) have enacted legislation related to newborn screening for CCHD; laws in 35 of those states (and the District of Columbia) require screening.

^†^ Thirty-one states issued regulations related to newborn screening; 15 of those states issued regulations requiring screening.

^§^ Implementation date refers to the date on which all birthing hospitals were expected to be screening, which might differ from the date when the health department implemented a screening policy or reporting requirement.

^¶^ Mandates CCHD screening of newborns.

** Direct entry midwives are midwives who typically attend home births and who have become credentialed without first becoming a nurse.

Forty-one (80%) jurisdictions reported receiving CCHD screening data from hospitals or birthing centers (Table 2). Among these jurisdictions, 32 (78%) receive some type of individual-level screening results for all infants screened, including 19 jurisdictions that receive all screening data (oxygen saturation values and dates and times of screening), one that receives only data on the final screen, and 12 that receive only the final interpretation result (pass/fail). Five (12%) of 41 jurisdictions reported receiving only aggregate data on the numbers of infants screened and CCHD cases detected, and four (10%) reported receiving individual-level screening results (oxygen saturation values and dates and times of screening) only for CCHD cases detected through screening.

**TABLE 2:** Receipt of critical congenital heart disease (CCHD) screening data and data sharing with birth defects surveillance programs — United States, 2018

Characteristic	No. (%) of jurisdictions
**Receipt of CCHD screening data by jurisdiction**	
Receive any CCHD screening data	41 (80)*
Receive any individual-level data	32 (78)^†^
Receive all individual-level screening data	19 (46)^†^
Receive individual screening data for CCHD cases only	4 (10)^†^
Receive data on final screen only	1 (2)^†^
Receive final pass/fail result	12 (29)^†^
Receive aggregate data only	5 (12)^†^
**Data sharing with birth defects surveillance systems**	
Data sharing exists	19 (37)*
**Mechanism of data sharing**	
Electronic linkage	5 (26)^§^
Shared data system	2 (11)^§^
Manual	12 (63)^§^
No data sharing	32 (63)*
**Reasons for no data sharing**	
No birth defects surveillance program	5 (16)^¶^
No individual level pulse oximetry screening data	10 (31)^¶^
Data systems not linked	17 (53)^¶^

* Percentage of all 51 jurisdictions (50 U.S. states and the District of Columbia).

^†^ Percentage of jurisdictions that receive any data.

^§^ Percentage of jurisdictions that share data.

^¶^ Percentage of jurisdictions that do not share data.

Nineteen (37%) jurisdictions reported data sharing between birth defects surveillance programs and newborn screening programs, maximizing the surveillance capabilities of these public health programs (Table 2). Shared data are used to identify cases of CCHD missed by screening, to ensure cases match between birth defects and newborn screening programs, or to perform postdiagnostic follow-up of infants identified by CCHD screening; six jurisdictions reported sharing for all three purposes. Among the 19 jurisdictions that reported data sharing, five had electronic linkage between newborn screening and birth defects surveillance data systems, two had a shared data system that encompasses both CCHD newborn screening and birth defects, and the remaining 12 shared data manually through direct communication, email, and reports. Among reasons cited by the 32 jurisdictions that do not share data between birth defects surveillance programs and CCHD newborn screening programs are absence of a birth defects surveillance program (five, 16%); lack of individual-level pulse oximetry screening data (10, 31%); and data systems that are not linked (17, 53%).

## Discussion

Policies for newborn screening of CCHD were gradually adopted in all U.S. states and DC from 2011 through 2018, thus facilitating improved survival of affected infants. Newborn screening mandates for CCHD have been found to save lives (*3*); however, opportunities continue for program improvement, particularly around data collection. Despite the implementation of CCHD screening policies in all jurisdictions, data collection efforts have lagged. In 2014, among 43 states that had implemented CCHD screening policies, 24 states were collecting data, although the types of data collected varied by state (*4*). By 2017, among 49 states with CCHD screening policies implemented, 41 were collecting data. Jurisdictional level data collection practices vary widely based upon state statute, financial and staff member resources, and capabilities to collect data (*8*). Completeness of data collection is important for surveillance, monitoring of outcomes, process improvement, and evaluation of state CCHD screening programs (*2*,*4*–*6*,*8*–*10*). States use screening algorithms as step-by-step guides for screening and determination of pass or fail and for the assessment of false positive and false negative cases (*6*,*9*). Evaluation and potential refinement of screening algorithms rely upon individual-level screening and outcome data.

Another opportunity for CCHD screening program evaluation and improvement lies in fostering collaborations between the two public health programs most invested in CCHD screening (newborn screening programs and birth defects surveillance programs). Because of the role of birth defects surveillance programs in monitoring new cases of CCHD, regardless of mode of detection, these programs have the ability to aid in evaluation of CCHD screening by assessing mortality, outcomes, and service utilization by children with CCHD (*8*). Integrating population-level screening and follow-up data from a CCHD newborn screening program with the targeted oversight of newly identified CCHD cases by birth defects surveillance programs is integral to establishing and maintaining a robust surveillance system. Ultimately, this integration can facilitate evaluation of the complete CCHD screening process, including the effectiveness of and adherence to the screening algorithm, screening sensitivity and specificity, and assessment of outcomes and needs of affected infants and their families. In Minnesota, for example, staff members of the CCHD newborn screening and birth defects surveillance program work together and share data regularly. Birth defects program and follow-up staff members have access to the same data system that collects individual-level CCHD screening data, facilitating rapid reporting of infants identified via CCHD screening to the birth defects surveillance program for diagnostic confirmation and connection to resources. Cases reported to the birth defects surveillance program also can be assessed easily for screening status and results, and previously undetected cases can be documented in the system.

The findings in this report are subject to at least two limitations. First, because of difficulty obtaining exact dates and interpretation of language in jurisdictions’ statutes and regulations, slight variability in the legislation, regulations, and guidelines presented might occur. Second, although all 51 jurisdictions completed the survey, the responses were reported by the jurisdictions’ CCHD screening contact person and not independently verified.

Newborn screening for CCHD in the United States has been implemented nationwide, with numerous infants’ lives being saved or improved as a result. Improved data collection practices and standardization across all jurisdictions could increase effective monitoring and evaluation of CCHD screening. Ongoing evaluation remains important to ensure the best possible outcomes.

**Summary**

**What is already known about this topic?**

Critical congenital heart disease (CCHD) occurs in two of every 1,000 births and might be undetected at birth. Affected infants are at risk for substantial morbidity and death early in life. In 2011, the U.S. Department of Health and Human Services Secretary endorsed the Advisory Committee on Heritable Disorders in Newborns and Children’s recommendation to add CCHD to the recommended universal newborn screening panel.

**What is added by this report?**

By 2018, all U.S. states and the District of Columbia had implemented newborn CCHD screening policies. Opportunities for program improvement, particularly around data collection, persist. Not all jurisdictions collect screening data or share data among relevant programs.

**What are the implications for public health practice?**

All U.S. newborns, regardless of which state they are born in, now have the opportunity to be screened for CCHD.
